# Darier disease is associated with heart failure: a cross-sectional case-control and population based study

**DOI:** 10.1038/s41598-020-63832-9

**Published:** 2020-04-23

**Authors:** Etty Bachar-Wikstrom, Philip Curman, Tara Ahanian, Ivone U. S. Leong, Henrik Larsson, Martin Cederlöf, Jakob D. Wikstrom

**Affiliations:** 1 Dermatology and Venereology Division, Department of Medicine (Solna), Karolinska Institutet, Stockholm, Sweden; 2 Dermato-Venereology Clinic, Karolinska University Hospital, Stockholm, Sweden; 3 Department of Medical Epidemiology and Biostatistics, Karolinska Institutet, Stockholm, Sweden; 4 Centre for Psychiatry Research, Department of Clinical Neuroscience, Karolinska Institutet, and Stockholm Health Care Services, Norra Stationsgatan 69, Stockholm, Sweden

**Keywords:** Skin diseases, Heart failure, Epidemiology, Genetics research

## Abstract

Human data supporting a role for endoplasmic reticulum (ER) stress and calcium dyshomeostasis in heart disease is scarce. Darier disease (DD) is a hereditary skin disease caused by mutations in the ATP2A2 gene encoding the sarcoendoplasmic-reticulum Ca2^+^ ATPase isoform 2 (SERCA2), which causes calcium dyshomeostasis and ER stress. We hypothesized that DD patients would have an increased risk for common heart disease. We performed a cross-sectional case-control clinical study on 25 DD patients and 25 matched controls; and a population-based cohort study on 935 subjects with DD and matched comparison subjects. Main outcomes and measures were N-terminal pro-brain natriuretic peptide, ECG and heart diagnosis (myocardial infarction, heart failure and arrythmia). DD subjects showed normal clinical heart phenotype including heart failure markers and ECG. The risk for heart failure was 1.59 (1,16-2,19) times elevated in DD subjects, while no major differences were found in myocardial infarcation or arrhythmias. Risk for heart failure when corrected for cardivascular risk factors or alcohol misuse was 1.53 (1.11–2.11) and 1.58 (1,15–2,18) respectively. Notably, heart failure occurred several years earlier in DD patients as compared to controls. We conclude that DD patients show a disease specific increased risk of heart failure which should be taken into account in patient management. The observation also strenghtens the clinical evidence on the important role of SERCA2 in heart failure pathophysiology.

## Introduction

Rare monogenic diseases provide opportunities to study human physiology and pathology from unique perspectives. Besides the importance of diagnosing and successfully treating patients with a rare disorder, major scientific breakthroughs resulting from investigation of rare diseases have often provided insight into more common disorders^1–3^. Herein, we examined increased risk of heart disease as a comorbidity of the rare skin disorder Darier disease (DD).

## Darier disease

Darier (Darier-White) disease (DD) was first described independently by Darier and White in 1889^4–6^. It is an autosomal-dominant skin disorder caused by mutations in the *ATP2A2* gene, which encodes for sarcoendoplasmic-reticulum Ca^2+^-ATPase isoform 2 (SERCA2)^7^. DD is characterized by a peculiar keratinization of the epidermis, nails, and mucus membranes resulting in a persistent hyperkeratotic papules^8^. The prevalence of the disease has been estimated to be 1 in 30,000–55,000 and the onset is usually before the third decade of life with complete penetrance in adults^9^. DD never remits, but oral retinoids may reduce hyperkeratosis. Interestingly, DD has been reported to be associated with epilepsy and neuropsychiatric problems including mild intellectual disability, bipolar disease and schizofrenia^10,11^.

## Role of SERCA2 in physiology and pathophysiology of the heart

In the healthy heart, Ca^2+^ is released from the sarcoplasmic reticulum (SR) via the ryanodine receptor 2 (RYR2), thus raising the cytosolic Ca^2+^ concentration and activating cardiac muscle contraction. The Ca^2+^ is then pumped back into the SR by SERCA2a lowering the cytosolic Ca^2+^ concentration to baseline levels and causing relaxation^12^. SERCA2 protein has three isoforms; SERCA2a, 2b and 2c. While SERCA2b is expressed ubiquitously in most tissues, SERCA2a is predominantly expressed in cardiomyocytes^12^. Heart failure (HF) is a highly morbid condition with increased incidence after the age of 65, affecting 1 in 100 individuals^13^. While efforts have been devoted to investigate the therapeutic value of various Ca^2+^ channels and proteins that regulate cytosolic Ca^2+^ concentrations, SERCA2a has received the most interest in recent years. Indeed, it was shown that the expression of SERCA2a is downregulated in HF, which subsequently contributes to severe systolic and diastolic dysfunction^14–16^. Consequently, restoring SERCA2a expression by gene transfer, both in HF patients^17,18^, as well as in animal models^19^, is elegantly described; however, with contradicting results. Thus, the role of SERCA2a is still unclear and further investigation is required.

In heart disease, pathological events such as overproduction of reactive oxygen species (ROS), ischemia/reperfusion, release of inflammatory cytokines and toxins, as well as perturbations in Ca^2+^ homeostasis can trigger endoplasmic reticulum (ER)/SR stress^20^. The latter is defined as an imbalance between the protein load and the folding capacity of the ER, which results in accumulation of misfolded proteins further activating an adaptive response termed the unfolded protein response (UPR). UPR is executed by a signaling network comprised of three ER-localized transmembrane proteins (IRE1, PERK and ATF6), which aim to restore the protein-folding homeostasis. Principally, for efficient cardiomyocyte contractile function, protein quality control is essential, and any perturbations in protein homeostasis can affect functionality and contractility of the myocytes, resulting in cell death^21–23^. Indeed, reduced SERCA2 expression or function causes the phenotype of HF also through induction of ER stress^24^.

With this background, we hypothesized that DD patients, who are characterized by ER Ca^2+^ dyshomeostasis and ER stress induced by endogenously reduced SERCA2 function^25–28^, are prone to heart failure.

## Methods

### Clinical study

#### Study design

A cross-sectional case-control study design was implemented in which we at one time-point measured cardiac function by heart failure markers N-terminal pro-brain natriuretic peptide (NT-proBNP), Galectin-3, suppression of tumorigenicity 2 (ST2), and troponin T as well as ECG and blood lipids (triglycerides, cholesterol including HDL and LDL). The rational for including troponin T was that it can be elevated in heart failure^29^ in addition to its use in detecting myocardial infarction.

#### Study group

The study included 25 patients with DD and 25 healthy volunteers matched by age, sex and body mass index (BMI). Age matching was made in ±5-year intervals and BMI was matched according to four categories: <18.5, 18.5–24.99, 25–29.99 and >30. During 2017–2018 DD subjects were recruited mainly from the Dermato-Venereology clinic at Karolinska University Hospital in Stockholm, Sweden, and through study participants since it is most often a familial condition. Control subjects were recruited through advertisements by e-mail and online as well as from a primary healthcare clinic in Stockholm. Inclusion criteria for the study were typical skin symptoms combined with histopathology-verified DD or family history of DD. Exclusion criteria were pregnancy, oral corticosteroids, recent acute illness (past 4 weeks), active substance abuse and severe kidney or liver disease. All patients but one was previously tested for *ATP2A2* mutations by whole exome and Sanger sequencing as previously described^30^.

#### Clinical assessment

Patients underwent a history taking focused on DD debut and family history. Family history was defined as having a first or second degree relative with DD. DD is most often treated with acitretin, an oral retinoid. Subjects who had been on oral acitretin treatment at some point during the 6 months prior to participation were classified into the acitretin category. Weight (kg) and height (cm) was measured to calculate BMI. The skin was inspected to verify the DD clinical diagnosis.

#### Collection of samples

Blood samples were collected after an overnight (10 h) fast. Since acitretin has a half-life of approximately 50 hours and is known to influence triglycerides and cholesterol^31–33^, subjects on oral acitretin discontinued the medication 7 days before the visit, longer wash-outs were not considered ethically.

#### Analytical techniques

Plasma NT-proBNP and troponin T was measured by immunochemistry and ElectroChemiLuminescence ImmunoAssay, and plasma triglycerides, cholesterol and HDL measured by enzymatic reaction and photometry (all using Cobas 6000, Roche Diagnostics Scandinavia AB, Stockholm, Sweden). Serum Galectin-3 and ST2 were determined using ELISA and photometry (Sunrise absorbance reader, Tecan Group Ltd., Männedorf, Zürich, Switzerland). LDL and HDL/LDL ratio were estimated from total cholesterol and HDL.

### Population-based cohort study

We conducted a cohort study based on linkage between several Swedish registers. The Total Population Register contains demographic information on all individuals registered as Swedish inhabitants since 1968. The National Patient Register^34^ contains discharge diagnoses for all inpatient care episodes since 1973, and for outpatient treatment since 2001. Diagnoses are assigned by physicians according to the WHO’s classification systems ICD-9 and ICD-10^35–37^. DD was defined by a diagnosis of the disorder according to the ICD-9 (757D) or ICD-10 (Q82.8E) in the National Patient Register. A total of 935 individuals with DD were identified. and matched with up to 100 comparison individuals selected randomly from the general population. Matching variables were birth year, sex and county of residence at the time of the first diagnosis in the DD index subject. Comparison individuals did not have a diagnosis of DD at the date of the first diagnosis of their respective index subjects with DD. Heart diseases were defined as a diagnosis of myocardial infarction (I21), heart failure (I50), or arrhythmia (I49) in ICD-10. While we are not aware of any validation of these specific diagnoses, most diagnoses of chronic diseases in the National Patient Register typically have positive predictive values between 85–95%^34^.

### Statistical analysis

In the clinical study descriptive analyses were performed to show frequencies, means, upper and lower limit and standard deviations. Since there were a small number of observations, Wilcoxon signed-rank test was used to compare continuous variables between groups. Differences between categorical variables were tested for significance using Fisher’s exact test. *P*-values < 0.05 were considered statistically significant. Demographic data (genetic background and presence of acitretin) were analyzed using two-way ANOVA and Bonferroni to correct for multiple comparisons. Data was analyzed by GraphPad Prism version 7.03 for Windows (GraphPad Software, La Jolla, CA, USA), and SPSS version 24.0 for Mac (SPSS, IBM, Armonk, NY, USA). The matched cohort design was used to examine associations between DD and heart disease. Odds ratios were estimated with conditional logistic regressions in SAS version 9.3 (SAS Institute, Cary, NC). In this design, odds ratios can be regarded as risk ratios, due to the incidence density sampling procedure. In additional analyses, we adjusted the results for the potential effects of cardiovascular risk factors of heart diseases, and alcohol misuse.

### Ethics

The study was approved by the Regional Ethics Committee in Stockholm and carried out in accordance with relevant guidelines and regulations such as the Helsinki declaration. All patients provided written confirmed consent for the clinical study.

## Results

### Patient baseline characteristics

Comparisons of baseline clinical parameters between subjects with DD and healthy control subjects are given in Table 1; no significant differences were found between cases and controls, except acitretin use, thus indicative of good matching. Two DD patients were adopted and heredity was unknown. There were no major differences in acitretin treatment between DD patients, sub-grouped by mutation type.

**Table 1:** Baseline characteristics of participants.

Baseline Characteristics	DD Patients	Control Patients	*P-Value*
*n*	25	25	
Age (years)	52 ± 13 (27–78)	51 ± 13 (27–76)	0,80^#^
Male sex (total number)	10 (40)	10 (40)	1,000^†^
BMI (kg/m^2^)	28,2±5,3 (18,9–42,3)	27,0±5,0 (19,8–38,7)	0,45^#^
Weight (kg)	81,4±17,8 (54–119)	78,8±18.2 (49,3–117)	0,73^#^
Height (cm)	169,6 ± 9,9 (152–193)	170,3 ± 11,5 (148,5–193)	0,83^#^
Current smoker	5 (20)	2 (8)	0,417^†^
DM family history	13 (52)	11 (44)	0,778^†^
Acitretin treatment	14 (56)	0 (0)	<0,001^†^
Hypertension treatment	3 (12)	4 (16)	1,000^†^
Dyslipidemia treatment	3 (12)	2 (8)	1,000^†^
**MutatioN related to treatment in DD patients (n** = **25)**	**Pathogenic Mutation**	**Benign/Unknown/** **No Mutation**	**Mutation Not Analyzed**
Acitretin treatment	9	4	1
Isotretinoin treatment	3	0	0
No systemic treatment	4	2	2
Retinoid treatment % of total	75%	67%	33%

Continuous variables were expressed as mean ± standard deviation (minimum-maximum). Other was expressed as number (total number) of criteria. Categorical values were expressed as a number (%).

^#^Mann-Whitney U Test; ^†^Fisher’s Exact Test; DD, Darier disease; n, number; BMI, body mass index; DM, diabetes mellitus.

### Heart failure biomarkers

We analysed several biomarkers for heart failure including the gold standard NT-proBNP as well as the novel heart failure biomarkers ST2 and Galectin-3^38,39^ (Table 2). Moreover, we measured Troponin T, which is a biomarker for cardiac necrosis in acute coronary syndrome; however, this can also be elevated in heart failure^29^. All these markers were within the normal range and with no significant differences between DD patients with or without mutation and control subjects (Table 2) and DD patients on acitretin did not show any significant differences (Supp. Table 1).

**Table 2:** Heart biomarkers in DD patients and controls sub-grouped for *ATP2A2* mutation.

	Control (n = 25)	Benign mutation (n = 9)	Pathogenic mutation (n = 15)
NT-proBNP (ng/L)	88,7 (11,0–954,0) ±186,6	64,0 (18,0–187,0) ± 55,4	126,4 (17,0–535,0) ±144,3
ST2 (µg/mL)	28,3 (12,7–48,2) ±9,7	28,4 (19,5–37,8) ± 6,2	32,3 (20,4–47,3) ± 6,8
Galectin-3 (µg/L)	14,7 (7,5–34,7) ±5,6	13,2 (9,5–20,5) ± 3,3	14,6 (9,2–18,3) ± 2,7
Trop T (ng/L)	5,4 (5,0–8,0) ± 1,1	7,0 (6,0–9,0) ± 1,1	9,0 (5,0–17,0) ± 4,1

Note that no significant differences were found. Data are reported as mean (Min-Max) ± SD. NT-proBNP, N-terminal pro-brain natriuretic peptide; Trop T, Troponin T; ST2, member of the interleukin 1 receptor family. Two-way ANOVA, Bonferroni were used for statistical analysis; however, there were no significant differences.

### Electrocardiogram (ECG)

A resting ECG was recorded to detect alterations in cardiac electrophysiology and analysed as shown in Table 3. While there was a significant increase above the normal upper range of 440 ms in QT-interval in DD patients with mutations, this was not found in frequency corrected QT-interval (QTc). However, among DD patients with acitretin treatment (irrespective of the mutation), there was a slight but statistically significant elevation of QT and QTc interval (Supp. Table 2).

**Table 3:** ECG parameters in DD patients and controls subgrouped for *ATP2A2* mutation.

	Control (n = 25)	Benign Mutation (n = 9)	Pathogenic Mutation (n = 15)
HR (bpm)	62,4 (46,0–84,0) ± 10,0	61,0 (50,0–78,0) ± 10,3	55,1 (47,0–80,0) ± 7,7
PQ Interval (ms)	159,0 (108,0–208,0) ± 24,0	150,3 (114,0–180,0) ± 22,3	145,9 (114,0–192,0) ± 19,9
QRS-Duration (ms)	93,5 (74,0–114,0) ± 9,7	96,0 (80,0–116,0) ± 12,9	102,9 (88,0–118,0) ± 7,7
QT Interval (ms)	414,7 (354,0–464,0) ± 26,4	433,78 (372,0–552,0) ± 55,0	453,1 (406,0–518,0) ± 34,3*
QTc Interval (ms)	419,4 (368,0–455,0) ± 22,8	433,3 (402,0–560,0) ± 48,9	431,9 (379,0–498,0) ± 33,8

Data are reported as mean (Min-Max) ± SD, in milliseconds (ms) or beats per minute (bpm). HR, heart rate. Two-way ANOVA, Bonferroni were used for statistical analysis.

*p < 0.001 control *vs* pathogenic mutation.

### Blood lipids

Blood lipids were measured due to their importance in heart disease and possible increase due to retinoid treatment^31–33^ (Table 4). Although within the normal interval there was a slight significant trend towards higher LDL/HDL ratio in DD patients with mutations and this was likely due to acitretin treatment as LDL/HDL ratio was also elevated in the group of DD patients with acitretin treatment (irrespective of the mutation) (Supp. Table 3).

**Table 4:** Blood lipid profile of DD patients and controls sub-grouped for *ATP2A2* mutation.

	Control (n = 25)	Benign Mutation (n = 9)	Pathogenic Mutation (n = 15)
LDL/HDL	2,1 (0,9–4,3) ± 1,0	2,4 (1,1–5,20) ± 1,4	3,1 (1,4–9,5) ± 1,9*
Triglycerides (mmol/L)	1,1 (0,5–3,9) ± 0,7	1,4 (0,6–3,1) ± 0,8	1,5 (0,8–2,8) ± 0,6
Cholesterol (mmol/L)	5,3 (3,6–7,0) ± 0,9	5,4 (3,3–7,9) ± 1,4	5,6 (4,2–7,7) ± 1,0
HDL (mmol/L)	1,7 (0,8–2,6) ± 0,5	1,5 (1,0–2,2) ± 0,4	1,4 (0,6–2,0) ± 0,4
LDL (mmol/L)	3,1 (2,0–5,1) ± 0,8	3,2 (1,6–5,4) ± 1,2	3,6 (2,6–5,7) ± 1,0

Data are reported as mean (Min-Max) ± SD. LDL, low- density lipoprotein; HDL, high-density lipoprotein. Two-way ANOVA, Bonferroni were used for statistical analysis.

*p < 0.001 Pathogenic mutation *vs* benign mutation.

### Association between Darier disease and common heart diseases

DD patients in general had a 59% higher risk of heart failure diagnosis, compared to undiagnosed matched individuals; risk ratio 1.59, 95% confidence interval 1.16–2.19 (Table 5). HF in DD patients occured at younger ages, 6.9 years earlier than in the comparison subjects. There was no conclusive evidence for increased risks of arrhythmia or myocardial infarcation. When these three heart diagnoses (heart failure, myocardial infarcation and arrythmia) were lumped together, the risk of any heart diagnosis was elevated with 32% in DD patients (men and women grouped); risk ratio 1.32, 95% confidence interval 1.02–1.70 (Table 5). It appeared as this increased risk of heart disease was mainly caused by women as these showed increased risk of heart disease (heart failure, myocardial infarcation and arrythmia) of 59%. The increased risk of HF (in men and women) was likely caused mainly by female risk increase as women showed increased risk of general heart disease however the study did not have enough power to conclude this. Moreover, as a number of risk factors are associated with development of cardiovascular disease and these may be overrepresented among DD patients we performed a second analysis in which cardiovascular risk factors were corrected for (Table 5). The main findings that heart failure is more common among DD patients and overall heart disease is more common among female DD patients were still significant (53% elevated risk).

**Table 5:** Risk of common heart diseases in DD (central illustration). Risk ratios (RR) and corresponding 95% confidence intervals (CI) expressing associations between heart disease diagnoses in individuals with Darier disease, compared with matched comparison individuals without Darier disease. Note that any heart diagnosis indicates all used heart diagnoses combined into one category. Note also that the total number of individuals with myocardial infarctions, heart failure or arrhythmia diagnoses was 97, while individuals with any heart diagnosis was 80, as some DD patients had more than one diagnosis. RR1; crude risk ratio. RR2; risk ratio adjusted for a lifetime diagnosis of alcohol misuse (ICD-10 code F10). RR3; risk ratio adjusted for a lifetime diagnosis of heart disease risk factors.

	Individuals with Darier disease (n = 935)	Matched comparison individuals (n = 93,487)	RR^1^ (CI)	P-value	RR^2^ (CI)	P-value	RR^3 §^ (CI)	P-value	Mean age in years at 1^st^ heart diagnosis in Darier patients/comparison individuals
	n (%)	n (%)							
I21 Myocardial infarction	29 (3,10)	2 828 (3,03)	1,03 (0,70–1,52)	0,88	1,03 (0,70–1,52)	0.89	0.95 (0.64–1.41)	0.80	65,9/71,9
I50 Heart failure	52 (5,56)	3 616 (3,87)	1,59 (1,16–2,19)	0,004	1,58 (1,15–2,18)	0.0041	1.53 (1.11–2.11)	0.009	70,0/76,9
I49 Arrythmia	15 (1,60)	1 330 (1,42)	1,13 (0,68–1,90)	0,64	1,12 (0,67–1,89)	0.65	1.10 (0.65–1.85)	0.72	67,9/61,4
Any heart diagnosis (*males & females*)	80 (8,56) **#**	6 504 (6,96)	1,32 (1,02–1,70)	0,04	1,31 (1,01–1,70)	0.04	1.26 (0.97–1.65)	0.09	
*Males*	36 (9,63)	3 431 (9,17)	1,07 (0,73–1,57)	0,74	1,06 (0,72–1,56)	0.76	1.10 (0.74–1.63)	0.65	
*Females*	44 (7,84)	3 073 (5,48)	1,59 (1,13–2,25)	0,008	1,59 (1,13–2,25)	0.008	1.46 (1.02–2.08)	0.04	

^#^Note that the total number of patients with either I21, I50 or I49 is higher than the total number of patients with any heart diagnosis as some patients had more than one diagnosis.

^§^Heart disease risk factors and ICD10 codes: Z72.0, Z72.0W (Tobacco use), Z72.0A (Smoking), I10.9 (Essential hypertension), I15.9 (Secondary hypertension, E10 (Type 1 diabetes), E11(Type 2 diabetes), E78.5 (Hyperlipidemia), E78.0 (Pure hypercholesterolemia), E78.1 (Pure hypertriglyceridemia), E78.2, E78.2×(Mixed hyperlipidemia), E78.0A (Familial hypercholesterolemia), E66.0, E66.8, E66.9 (Obesity).

## Discussion

While our clinical study found no heart abnormalites in DD patients our population study indicates that DD patients are more prone to develop HF and at an earlier age. Possible reasons behind the observed excess risk of HF in DD are outlined below.

### SERCA2 and heart disease

DD is mainly caused by a mutation in the *ATP2A2* gene^7^, which encodes for the SR/ER Ca^2+^ pump- SERCA2. To better understand and further characterize the consequenses of SERCA2 dysfunction in DD patients, we searched for more common conditions in which SERCA2 plays a central role and chose to focus on heart disease, as the importance of SERCA2 in heart function is well established. In both animal and human heart failure, SERCA2a expression is significantly decreased, which leads to abnormal Ca^2+^ handling and a deficient contractile state and HF^40^. Interestingly, SERCA2 haplosufficent mice were reported to develop HF when crossed with a with a transgenic model of increased myofibrillar Ca^2+^-sensitivity^41–43^, thus suggesting that DD patients may be more susceptible to HF. Further, in attempts to rescue SERCA2a deficiency phenotype, a gene-targeted mouse with full substitution of the cardiac isoform SERCA2a by SERCA2b was described^44^. The 2b isoform has a nearly 2-fold higher affinity for Ca^2+^ but a lower maximal catalytic turnover rate than 2a, which resulted in a mild concentric heart hypertrophy and impaired contraction-relaxation. This suggests that also dysfunctional and not only defiency in SERCA2 may cause HF. Importantly, *Atp2a2*^−/+^ mice develop squamous cell carcinomas but no DD-like lesions, thus mouse SERCA2 pathophysiology may therefore differ substantially from humans^45^. Our study presents the first evidence that HF is accociated with DD which adds to the growing body of evidence that DD is a systemic condition not only confined to the skin. This finding also adds a novel angle to understanding of SERCA2 pathophysiolog in human HF.

### Gender differences in heart failure

Our findings indicates that DD female patients have higher risk for heart disease in general and probably specifically for heart failure. In fact, over the past decade, one of the most robust findings across numerous HF studies was a distinct difference in gender distribution. Namely, women significantly outnumber men, leading to a gender ratio of approximately 2:1 in HF, with preserved ejection fraction^46,47^. While we do not know what subtype of HF is overrepresentend in DD female patients, these gender differences in HF may be explained by the presence of dysfunctional SERCA2 as it has been shown in multiple separate studies that increased oxidative stress observed in damaged myocardium lead to impairment of the SERCA pump followed by Ca^2+^ dyshomeostasis and prolonged diastolic relaxation, which contributed to the females’ predominent heart failure type^41–43^.

### Confounding factors

An alternative explanation to the DD-HF correlation might be the long term usage of acitretin to treat DD, that makes these patients more susceptible to HF. Acitretin is a vitamin A derivative and a second generation retinoid which has anti-inflammatory and immunomodulatory effects^48^. Among the side effects associated with this drug, it has been reported that treatment with acitretin increases serum lipids and triglycerides in 25–50% of patients^49,50^, however the DD-HF association did not change significantly after correction for cardiovascular risk factors including hyperlipidemia and hypertriglyceridemia (Table 5). Another possible explanation for our DD-HF findings may lie in the fact that chronic skin disease by itself may increase the risk of cardiovascular disease^51^. Because the national registers in the cohort study lacked potentially important individual lifestyle characteristics, the results from these analyses warrant replication in a more informative data set where statistical adjustment for such factors are possible.

## Conclusions

DD is likely a syndrome with multi-organ involvement. The association between DD and HF we demonstrated here can be further understood in several ways. Most important will be to characterize what kind of heart failure DD patients have, in order to tailor treatment. Careful assessment of heart function in healthy individuals would further increase our understanding of SERCA2 in normal heart physiology. Importantly, it may be that DD would require systemic treatment from early age to combat multi-organ involvement however before that can be realized we must further map the its’ organ involvement. Moreover, there is currently no therapy available for such treatment.

### Clinical perspectives

The increased risk of heart failure should be considered in patients with DD that although being a rare disease affects around 200,000 patients worldwide. While speculative, it may be that heart failure in DD show special characteristics.

### Translational outlook

This study adds significant human evidence for a role of SERCA2 in heart failure and adds to the growing body of literature suggesting SERCA2 as a potential terapeutic target.

## Supplementary information

Supplementary Information.

## Data Availability

The datasets generated during and/or analysed during the current study are available from the corresponding author on reasonable request.
