# Supplementary material for: Darier disease is associated with heart failure: a cross-sectional case-control and population based study
Source: Sci Rep. 2020 Apr 23;10:6886. doi: 10.1038/s41598-020-63832-9 (PMC7181854; doi:10.1038/s41598-020-63832-9)
Supplement: Supplementary file 1 — Supplementary Information. [file 41598_2020_63832_MOESM1_ESM.docx]

**Supplemental data**

**Title:** **Darier disease is associated with heart failure: a cross-sectional case-control and population based study**

Etty Bachar-Wikstrom PhD^a^, Philip Curman MD^a,b^, Tara Ahanian MD^a,b^, Ivone U. S. Leong PhD^a^ , Henrik Larsson PhD^c^, Martin Cederlöf PhD^d,^ Jakob D. Wikstrom MD PhD^a,b^*

a Dermatology and Venereology Division, Department of Medicine (Solna), Karolinska Institutet, Stockholm, Sweden

b Dermato-Venereology Clinic, Karolinska University Hospital, Stockholm, Sweden.

c Department of Medical Epidemiology and Biostatistics, Karolinska Institutet, Stockholm, Sweden

d Centre for Psychiatry Research, Department of Clinical Neuroscience, Karolinska Institutet, and Stockholm Health Care Services, Norra Stationsgatan 69, Stockholm, Sweden

**Supplemental Table 1. Heart biomarkers in DD patients and controls sub-grouped for acitretin treatment.**

|  | CONTROL  (n = 25) | DARIER – ACITRETIN  (n = 14) | DARIER - NON ACITRETIN  (n = 11) |
| --- | --- | --- | --- |
| NT-proBNP (ng/L) | 88,7 (11 – 954)  ± 186,6 | 119,6 (17 - 535)  ± 152,3 | 80,7 (18 – 187)  ± 52,2 |
| ST2  (µg/mL) | 28,3 (12,7 - 48,2)  ± 9,7 | 30,4 (20,4 - 37,8)  ± 6,1 | 31,3 (19,5 - 47,3)  ± 7,5 |
| Galectin-3 (µg/L) | 14,7 (7,5 - 34,7)  ± 5,6 | 15 (11 - 20,5)  ± 2,4 | 12,7 (9,2 - 18,3)  ± 3,1 |
| Trop T  (ng/L) | 6 (5 – 8)  ± 1,1 | 7 (5 – 17)  ± 8,7 | 8 (6 – 10)  ± 7,8 |

Note that no significant differences were found. Data are reported as mean (Min-Max) ± SD. NT-proBNP, N-terminal pro-brain natriuretic peptide; Trop T, Troponin T; ST2, member of the interleukin 1 receptor family. Two-way ANOVA, Bonferroni were used for statistical analysis; however, there were no significant differences.

**Supplemental Table 2. ECG parameters in DD patients and controls subgrouped for acitretin treatment.**

|  | CONTROL  (n = 25) | DARIER – ACITRETIN  (n = 14) | DARIER - NON ACITRETIN  (n = 11) |
| --- | --- | --- | --- |
| HR (bpm) | 62,4 (46 – 84) ± 10,0 | 57,4 (50 – 80) ± 9,3 | 58,2 (47 – 78) ± 9,1 |
| PQ Interval (ms) | 159 (108 – 208) ± 24,0 | 150,9 (114 – 192) ± 20,9 | 146,2 (114 – 180) ± 21,8 |
| QRS-Duration (ms) | 93,5 (74 – 114) ± 9,7 | 101 (82 – 118) ± 9,3 | 98,6 (80 – 118) ± 11,6 |
| QT Interval (ms) | 414,7 (354 – 464) ± 26,4 | 454,9 (378 – 552) ± 45,5 **#** | 431,8 (372 – 504) ± 36,5 |
| QTc Interval (ms) | 419,4 (368 – 455)± 22,8 | 442 (379 – 560) ± 48,2 **#** | 421,3 (404 – 446) ± 15,2 |

Data are reported as mean (Min-Max) ± SD, in milliseconds (ms) or beats per minute (bpm). HR, heart rate. Two-way ANOVA, Bonferroni were used for statistical analysis.

**#** p<0.001 control vs Darier- Acitretin.

**Supplemental Table 3. Blood lipid profile of DD patients and controls sub-grouped for acitretin treatment.**

|  | CONTROL  (n = 25) | DARIER – ACITRETIN  (n = 14) | DARIER – NON ACITRETIN  (n = 11) |
| --- | --- | --- | --- |
| LDL/HDL | 2,1 (0,9 - 4,3) ± 1,0 | 3,2 (1,6 - 9,5) ± 2,1**#** | 2,3 (1,1 - 3,8) ± 0,9 **^§^** |
| Triglycerides (mmol/L) | 1,1 (0,5 - 3,9) ± 0,7 | 1,6 (0,8 - 3,1) ± 0,7 | 1,5 (0,56 - 2,9) ± 0,8 |
| Cholesterol (mmol/L) | 5,3 (3,6 – 7) ± 0,9 | 5,8 (4,6 - 7,9) ± 1,1 | 5,2 (3,3 - 7,1) ± 1,2 |
| HDL  (mmol/L) | 1,7 (0,8 - 2,6) ± 0,5 | 1,3 (0,6 – 2) ± 0,4 | 1,5 (1 - 2,2) ±0,4 |
| LDL  (mmol/L) | 3,1 (2 - 5,1) ± 0,8 | 3,8 (2,6 - 5,7) ± 1,1 | 3,1 (1,6 - 4,6) ± 0,9 |

Data are reported as mean (Min-Max) ± SD. LDL, low- density lipoprotein; HDL, high-density lipoprotein. Two-way ANOVA, Bonferroni were used for statistical analysis.

**#** p<0.001 control vs Darier- Acitretin; **§** p<0.001 Darier - Acitretin vs Darier – Non Acitretin.
